# Escherichia coli-associated Infective Endocarditis in a Patient with Septic Abortion: A Rare Culprit in a Unique Presentation

**DOI:** 10.7759/cureus.5632

**Published:** 2019-09-12

**Authors:** Zauraiz Anjum, Zemal Tariq

**Affiliations:** 1 Internal Medicine, Fatima Jinnah Medical University, Lahore, PAK; 2 Internal Medicine, Gujranwala Medical College, Gujranwala, PAK

**Keywords:** infective endocarditis, gram-negative bacteria, gram-negative rods, non-hacek, escherichia coli, septic abortion

## Abstract

Infective endocarditis (IE) is a condition characterized by the infection of the endocardium of the heart. The endocardium can include a heart valve or mural endocardium. IE has several known pathogens, including Streptococcus viridans, Staphylococcus aureus, and HACEK organisms (i.e., Hemophilus species, Actinobacillus actinomycetemcomitans, Cardiobacterium hominis, Eikenella corrodens, or Kingella species). In this report, we present a case of a young woman presenting with IE with Escherichia coli following a septic abortion. This case highlights IE with E. coli as a rare but potentially devastating complication of septic abortions, especially those associated with septicemia.

## Introduction

Infective endocarditis (IE) can be caused by different pathogens, including, but not limited to *Streptococcus viridans*, *Staphylococcus aureus*, and HACEK organisms (i.e., Hemophilus species, Actinobacillus actinomycetemcomitans, Cardiobacterium hominis, Eikenella corrodens, or Kingella species) [1]. IE can also be a complication of systemic lupus erythematosus.

Septic abortion leading to *Escherichia coli* sepsis, subsequently leading to IE in an otherwise healthy, young heart is very rare [1-5]. There are few reported cases of IE due to *E. coli*; fewer still were preceded by urosepsis [6]. There are no cases published in the literature of IE by *E. coli* following a septic abortion. This case study and following literature review highlights the above-mentioned rare complication, so that appropriate and timely measures can be taken by physicians to avoid potentially fatal outcomes.

## Case presentation

A 21-year-old woman presented to the ED after a septic abortion. She was managed by the gynecological team in the ED and was later shifted to the medical floor. After stabilization, she was discharged home but presented three weeks later with fever, nonproductive cough, swollen feet, orthopnea, and exertional shortness of breath. On further inquiry, she claimed that she had felt feverish for a few days following her discharge. She denied any facial swelling or shortness of breath on exposure to pollen/seasonal change/dust or smoke. She further denied any abnormality in her echocardiogram or electrocardiogram (EKG) done per routine prepregnancy or any other systemic symptoms like nausea, vomiting, diarrhea, joint pain, bowel or bladder symptoms.

Her physical examination revealed a blood pressure of 110/70 mmHg, pulse rate of 102 beats per minute, respiratory rate of 21 breaths per minute, and oxygen saturation of 97%. She appeared agitated. She had bilateral, grade two pitting edema of her ankles. On cardiovascular examination, there was a new systolic murmur over the apex. We noted no gallops or rubs. On auscultation, her chest had bilateral crackles, especially on the right side. The results of her abdominal and neurological examinations were unremarkable. Her past medical, social, sexual, or family history was not significant.

**Figure 1:**
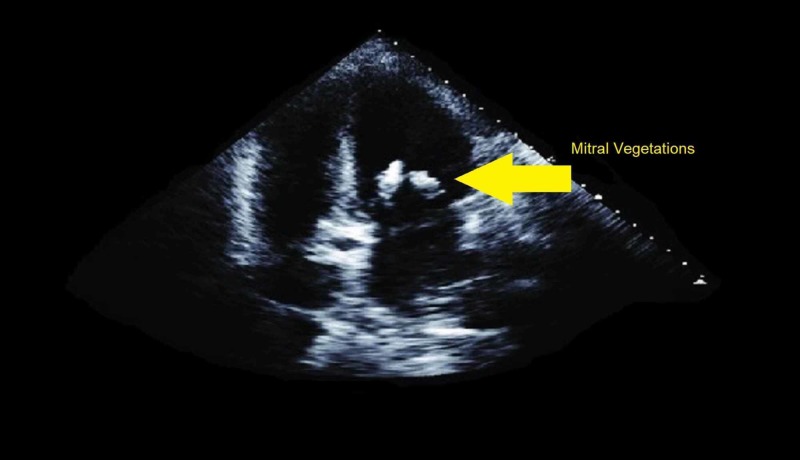
TTE of the patient showing mitral vegetations. TTE, transthoracic echocardiography

Her pertinent laboratory findings at this time revealed a WBC count of 28.2/µL; her creatinine was 1.8 mg/dL, and her hemoglobin was 10.6 g/dL. Transthoracic echocardiography (TTE) revealed vegetation at the mitral valve leaflets (see Figure 1).

**Figure 2:**
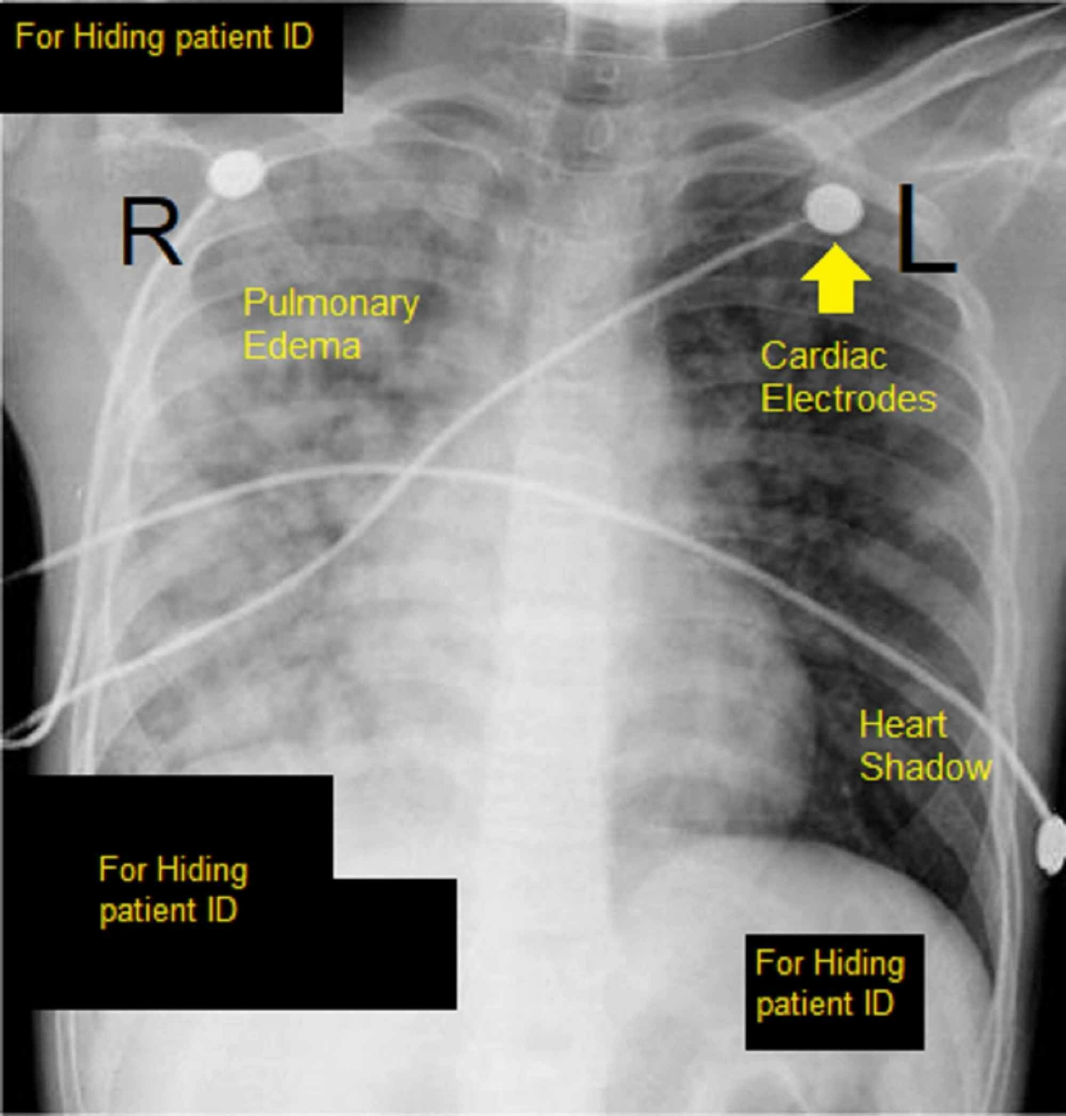
CXR showing pulmonary edema. CXR, chest X-ray

The TTE also revealed a flail mitral valve with regurgitation. Her electrocardiogram appeared normal. Her chest X-ray (CXR) revealed signs of pulmonary edema (see Figure 2).

Both aerobic and anerobic blood cultures were sent to the hospital laboratory for evaluation. The patient was given paracetamol to take as needed, and IV furosemide 40 mg (twice daily). She was shifted to the cardiology floor for further management and observation. The blood cultures revealed *E. coli* as a possible cause, and sensitivity showed them to be susceptible to ciprofloxacin and amikacin. The patient was started on oral ciprofloxacin 500 mg twice daily and amikacin in an adjusted renal dose. In addition, she was given iron supplements to help with her anemia. During her stay, she had an episode of right-sided weakness lasting 39 minutes and was started on anticoagulation therapy.

Owing to persistently elevated WBCs, fever, and lack of improvement in her condition, she was chosen for, consulted on, and consented to valve replacement; the replacement procedure was performed on day 12 of her second stay. A 27-mm St. Jude Medical Biocor™ prosthesis (St. Jude Medical, Inc., St Paul, MN, USA) was used. Postprocedural TTE revealed that mitral regurgitation had resolved. Her symptoms began improving, and ultimately, diuresis and antipyretics were stopped.

Our patient was diagnosed using the modified Duke criteria for endocarditis. Our diagnosis was further strengthened by the positive intra-operative findings. The patient had no valvular defect or co-morbidity that may have led to this outcome, and the only causative factor was the septic abortion.

The causes of persistent fever despite appropriate management of the suggested culprit are extensive. Our patient had persistent fever and septicemia despite appropriate management of the apparent gynecological and medical cause. The patient had no indwelling catheters or indication of an infection with a super-bug. Furthermore, the patient had a healthy mitral valve and even more interestingly, no comorbidity.

She was subsequently discharged in a stable condition and advised to report for an outpatient follow-up evaluation. Ciprofloxacin was continued for six weeks and amikacin for four weeks. Two consecutive subsequent blood cultures were sterile. She denied any new active concerns and reported good compliance with medical advice and resolution of the previous symptoms.

## Discussion

Infective endocarditis is a well-known condition involving infection of heart endocardium, especially heart valves, and is a deadly condition associated with high mortality [1]. IE caused by Gram-negative rods is very rare. One study reported 961 cases of endocarditis, among which 24 cases (2.5%) were caused by Gram-negative bacilli. *E. coli*, *Pseudomonas aeruginosa*, and *Salmonella enterica* turned out to be the most common organisms involved [2]. The most commonly encountered setting was a native valve (85.7%), and most of them were damaged (57%) [2].

In a six-year study of cases of Gram-negative endocarditis in a French hospital, only 17 cases of definite Gram-negative IE were noted; 12 cases (70%) were due to non-HACEK organisms, and five cases (30%) were due to HACEK organisms. The non-HACEK group involved *E. coli* (4/12) and *P. aeruginosa* (3/12 patients) as the main culprits. Most cases (10/17) were associated with a prosthetic valve (eight in the non-HACEK and two in the HACEK group) [3].

One study found *E. coli*-associated IE in patients who had a recent endoscopic retrograde cholangiopancreatography [4], and another study found *E. coli*-associated IE in a patient of liver transplantation [5].

Today, the patients with *E. coli*-associated IE commonly have diabetes and are generally older than the patient population born before 1960 [1]. The prosthetic valves are usually involved, and the source of infection is usually the urinary tract [1]. Regarding IE, Noureddine et al. found Gram-negative bacilli affected people with comorbidities more often compared to other common organisms (90% vs. 39%) [2]. Diabetes, hepatic cirrhosis, and neoplasms were the most common comorbidities. In that study, 47.6% of the cases had evidence of previous intervention, and hospital-acquired infections occurred in 37% of the cases [2]. Common complications of IE due to Gram-negative bacteria are renal failure (41%), central nervous system involvement (33%), and ventricular dysfunction (45%) [2].

The mortality rate associated with Gram-negative bacteria, although lower compared to that in patients born before 1960, is still significantly high, and the patients commonly require surgery [1]. Cardiac surgery was necessary for 21% of patients due to ventricular dysfunction, according to Noureddine et al. [2]. Aminoglycosides were tried in more than half of the cases, but no improvements were noted. Both surgery and combination antibiotic therapy had no significant survival benefit [6]. IE associated with other organisms had lower mortality than the one related to Gram-negative organisms (35% vs. 41%) [2].

## Conclusions

Our case report suggests that persistent fever should prompt an echocardiogram as it could be a clue to a cardiac infection. A prompt multidisciplinary, collaborative approach involving medical, cardiologic, and cardiovascular surgery teams is necessary to save such a patient.
